# Adult‐onset Niemann–Pick disease type C masquerading as spinocerebellar ataxia

**DOI:** 10.1002/mgg3.1906

**Published:** 2022-02-22

**Authors:** Mary L. Vo, Tess Levy, Shenela Lakhani, Chengbing Wang, M. Elizabeth Ross

**Affiliations:** ^1^ Department of Neurology Weill Cornell Medicine New York New York USA; ^2^ Center for Neurogenetics Feil Family Brain and Mind Research Institute, Weill Cornell Medicine New York New York USA; ^3^ Present address: Icahn School of Medicine at Mt. Sinai New York New York USA

**Keywords:** adult‐onset, cerebellar ataxia, Niemann‐Pick disease, NPC, whole exome sequencing

## Abstract

**Background:**

Adult‐onset Nieman–Pick disease type C (NPC) is a rare progressive ataxia caused by lysosomal accumulation of unesterified cholesterol resulting in severe disability and death. The diagnosis of NPC can be challenging as clinical features overlap with other more common hereditary ataxias. This study pursued the molecular genetic basis of adult‐onset cerebellar ataxia manifesting in two siblings. A prior diagnosis of spinocerebellar ataxia type 2 (SCA2) based on an ataxia gene panel was questioned when the younger sibling developed similar symptoms but had discordant genetic results.

**Methods:**

Neurologic examination, whole exome sequence (WES), targeted sequence to establish genome phasing, and cytochemical and biochemical studies of fibroblast cultures were employed.

**Results:**

The pedigree and neurological examinations suggested a recessive or possibly dominant cerebellar ataxia. WES showed the siblings were both compound heterozygous for two rare variants in the *NPC1* gene—one pathogenic, stop gain at p.Arg934Ter (NM_000271.4), and a missense change, p.Pro471Leu (NM_000271.4), of uncertain significance. Filipin staining of fibroblast cultures showed lysosomal cholesterol accumulation and biochemical assay demonstrated impaired cholesterol esterification.

**Conclusions:**

The study established the correct molecular diagnosis of biallelic, adult‐onset NPC in a patient initially diagnosed with SCA. Additionally, the p.Pro471Leu variant was identified as likely pathogenic. Inaccurate molecular diagnosis will deprive NPC patients of treatment options. Investigation using WES is justified when a detected expansion size is in the borderline range for pathogenicity.

## Graphical abstract

We present a sibling pair who were initially diagnosed with adult‐onset spinocerebellar ataxia Type 2 based on borderline repeat expansion in one sibling. Whole exome sequencing revealed two rare variants including the novel variant p.Pro471Leu associated with NPC. Biochemical testing in both siblings confirmed the diagnosis of NPC, a treatable neurovisceral disease.

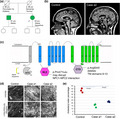

## 1 INTRODUCTION

Niemann–Pick disease type C (NPC) is a rare, fatal autosomal recessive neurovisceral disorder caused by lysosomal accumulation of unesterified cholesterol. The classic childhood form features cerebellar ataxia, supranuclear gaze palsy, dysarthria, developmental regression, and neuropsychiatric and metabolic disturbances with a life expectancy of 12 years (Garver et al., 2007). The adult form is widely heterogeneous and can present with cerebellar ataxia, dysarthria, dysphagia, spasticity, dystonia, seizures, vertical supranuclear gaze palsy (Piroth et al., 2017), parkinsonism, cognitive impairment, and psychosis (Sévin et al., 2007). Hepatomegaly and splenomegaly are often absent in adult‐onset cases. The clinical course is progressive, ultimately leading to death if untreated.

More than 95% of NPC cases are associated with loss‐of‐function variants in *NPC1* or *NPC2* (Dardis et al., 2020). Increased utilization of whole exome sequencing (WES) has accelerated the diagnostic frequency of the adult form to an incidence between 1:19,000 and 36,000 (Wassif et al., 2016). Biochemical diagnosis utilizes patient fibroblasts in an assay to demonstrate delayed LDL‐cholesterol esterification and filipin staining to show intracellular accumulation of unesterified cholesterol (Geberhiwot et al., 2018).

The rarity of adult NPC and lack of defined phenotypic spectrum make the clinical diagnosis challenging. Moreover, adult‐onset NPC possesses many overlapping clinical features with more common hereditary ataxic disorders and is likely underrecognized. We identify a sibling pair with adult‐onset NPC found to have compound heterozygous mutations of NPC.

## 2 METHODS

### 2.1 Genetic testing

In view of her sibling’s reported diagnosis of SCA2, the proband’s sample was submitted to Athena Diagnostics for an ataxia panel that included trinucleotide repeat testing of ATXN2. Blood samples from both siblings were submitted to GeneDx for WES. After exon capture, DNA libraries were synthesized and subjected to massively parallel sequencing on an Illumina platform to generate paired end reads that were aligned based on NCBI Refseq transcripts and genome build GRCh37/UCSC hg19. Clinically significant variants were verified using an orthogonal method. Mean depth of coverage was 81X and 83X for proband and sibling, respectively, with at least 98.5% of exons covered at a minimum of 10X. Variants were phased through targeted DNA sequencing at GeneDx of NPC1 performed on a buccal sample from the proband's son.

### 2.2 Fibroblast cultures

After obtaining informed consent, patient and control fibroblast cultures were generated from 3 mm dermal punch biopsies using standard procedures. Primary skin fibroblasts were cultured in modified eagle's medium (MEM, GIBCO) supplemented with 20% fetal bovine serum (FBS, GIBCO) and 1 mM penicillin–streptomycin. All experiments were performed with fibroblasts at passage 2–5.

### 2.3 Biochemical analysis

Cells from patient skin biopsies were propagated in 20% FBS. The immortalized skin fibroblast cells were derived from the propagated cells and cultured in 10% FBS at low passages. Confluent fibroblast cultures were incubated for 48 hours in DMEM supplemented with 10% lipoprotein‐deficient serum (LPDS, 880100‐5, Kalen Biomedical, Germantown, MD). After 2 days, cells were switched to DMEM +10%LPDS + purified LDL (50 μg protein/ml medium, L7914, MilliporeSigma) for 24 h. Intracellular cholesterol was visualized by filipin staining per established protocol (Vanier & Latour, 2015). Cholesterol esterification in cells was measured using the Cholesterol/Cholesteryl Ester Quantitation Assay Kit (Colorimetric/Fluorometric assay, ab65350, Abcam) according to manufacturer’s protocol. The total cholesterol and free cholesterol in cell extracts were measured separately. Cholesteryl esters = Total cholesterol ‐Free cholesterol. Ester ratio = Cholesteryl esters/Total cholesterol × 100%. Each experiment was performed in triplicate and a student's *t*‐test was used to determine statistical significance at *p* < 0.01.

## 3 RESULTS

**FIGURE 1:**
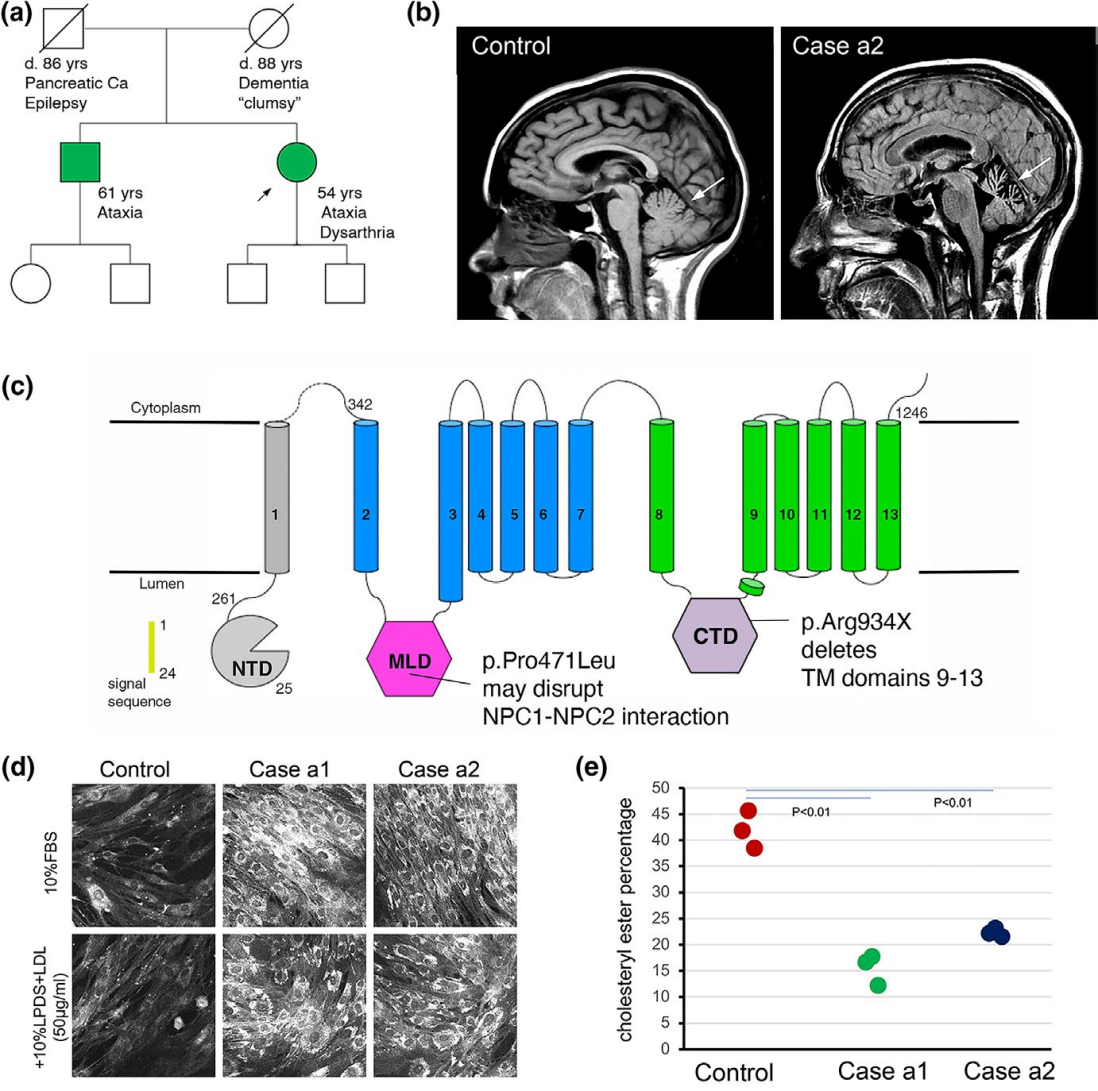
Adult sibling pair affected with cerebellar ataxia. (a) Pedigree (proband indicated with arrow). (b) MRI of one sibling (AR18054a2) showing cerebellar degeneration (white arrow). (c) Cartoon of NPC1 protein with identity and location of the pathogenic alleles. d. Patient fibroblasts showing lipid accumulation within cells (Filipin staining). (e) Defective cholesteryl esterification in affected sibs. *n* = 3 biological replicates. CTD, C‐terminal domain; MLD, middle luminal domain; NTD, N‐terminal domain; TM, transmembrane domain; WT, wildtype control. NPC1 structure taken from Li et al. (2016)

The siblings were offspring of non‐consanguineous parents and displayed symptoms consistent with an inherited, autosomal recessive or autosomal dominant cerebellar disorder with variable penetrance (Figure 1a). The proband, Patient a1, was a 55‐year‐old woman who presented to the WCM Neurogenetics Clinic with 1 year of progressive imbalance, ataxia and dysarthria. The neuro‐ophthalmologic examination showed slowed and incomplete vertical saccades with full range of motion during vestibulo‐ocular reflex (VOR) testing, subtle delayed pursuit in all directions, and rare saccadic intrusions without nystagmus. The neurological exam was significant for dysarthria, appendicular ataxia, and wide‐based, ataxic gait. Cognitive, motor, and reflex examinations were normal. Her brother, Patient a2, was previously diagnosed with adult‐onset spinocerebellar ataxia type 2 (SCA2). Her mother had had clumsiness and frequent falls, but no formal evaluation was pursued. Genetic testing of the proband for SCA2 was normal with 22 and 22 *ATXN2* repeats (normal range ≤ 31 repeats).

The proband's brother, Patient a2, was a 61‐year‐old man who developed ataxia, syncopal episodes, and gait instability in his 40s and was unable to continue working by age 48 (Figure 1a). His examination at age 64 was notable for vertical gaze palsy without nystagmus, mild dysarthria, moderate dysmetria in all extremities. He had a wide‐based, ataxic gait and required a rolling walker. He had distal gradient sensory loss and areflexia that was attributed to diabetic polyneuropathy. Mental status testing, motor examination, and tone were normal. Neuropsychiatric symptoms were denied. MRI of brain (Figure 1b) showed prominent cerebellar atrophy. Genetic testing on an ataxia panel identified a CAG repeat size in *ATXN2* of 32 and 22 repeats, confirmed on repeat testing. Patient a2 was previously given a diagnosis of autosomal dominant SCA2 on that basis. Neither sibling had LDH abnormalities or hepatomegaly.

Clinical WES for both siblings showed two variants in NPC1 (Figure 1c). NM_000271.5:c.2800C > T (p.Arg934Ter) was a nonsense variant predicted to result in loss of function through protein truncation or nonsense mediated decay. p.Arg934Ter is not observed at a significant frequency in large population cohorts. The other, NM_000271.5:c.1412C > T (p.Pro471Leu), was a missense variant of uncertain significance (VUS). p.Pro471Leu has a very low allele frequency across all populations (<0.001). Interestingly, missense mutations producing conformational changes in the middle luminal domain (MLD) of the NPC1 glycoprotein can alter the relative position of the middle and C‐terminal luminal domains and disrupt the cholesterol efflux tunnel in experimental models (Vanharanta et al., 2020). Mutations affecting the MLD have been shown to interfere with *NPC1* interactions with other proteins, including *NPC2* (Li et al., 2016). The short DNA sequence reads of WES could not distinguish these variants as residing on the same (in cis) or both autosomes (in trans). Targeted sequencing of *NPC1* in the proband's unaffected adult son showed that c.2800C > T and c.1412C > T occurred in trans, confirming the proband and her brother to be biallelic for these *NPC1* variants, a necessary condition for an autosomal recessive disorder.

Biochemical testing on patient fibroblasts revealed intracellular accumulations of unesterified cholesterol seen on filipin staining (Figure 1d). In addition, cholesterol esterification was impaired in fibroblasts from both siblings compared to an age‐matched healthy control (Figure 1e). Results of genetic sequencing combined with biochemical tests facilitated recharacterization of the *NPC1* p.Pro471Leu variant as likely pathogenic, indicating the diagnosis of biallelic *NPC1*. Both siblings were offered treatment with miglustat.

## 4 DISCUSSION

We report a sibling pair with adult‐onset cerebellar ataxia caused by compound heterozygous *NPC1* variants including a novel variant, p.Pro471Leu, occurring with a known pathogenic variant, p.Arg934Ter.

This observation again points out the importance of WES in the workup of adult‐onset ataxia, as singleton cases of adult cerebellar ataxia are often presumed to result from an autosomal dominant mutation and may be considered resolved if a suggestive result returns from an SCA gene panel. Inclusion of WES in neurogenetic evaluation should be obtained when a diagnostic panel indicates a possible but not definitive diagnosis.

The proband’s brother was initially misdiagnosed SCA2 on the basis of a borderline CAG repeat expansion in *ATXN2*. SCA2 presents with ataxia, hyporeflexia, areflexia, slow saccades, and rarely dementia. Symptom presentation is highly variable in patients harboring ~31 CAG repeats. One study of SCA families showed that repeat expansion numbers correlate inversely with age of onset (35 repeats = 74 years, mean onset) (Giunti et al., 1998). We cannot exclude the possibility that borderline SCA repeat expansion might further contribute to the severity of the phenotype in the Patient a2.

Suspicion of NPC should be raised for adult patients presenting with ataxia and vertical supranuclear gaze palsy (VSGP), especially with cognitive impairment and psychiatric symptoms, to prompt further genetic and biochemical investigation (Geberhiwot et al., 2018). Miglustat has been shown to halt or slow progression of neurologic symptoms in patients with pre‐symptomatic or mildly symptomatic NPC (Pineda et al., 2018) by stabilizing or improving oculomotor function, swallowing, ambulation, and cognition. Miglustat is an iminosugar that reversibly inhibits glucosylceramide synthase and reduces glycosphingolipid synthesis (Giunti et al., 1998).

Increasing availability of commercial genetic testing has expanded appreciation that adult‐onset neurodegenerative disorders may include conditions more typically encountered in children. However, intermediate repeat expansion and VUS results must be interpreted with caution and require the expertise of comprehensive genetics teams able to pursue variant analysis, extend bioinformatic analysis, access international databases of emerging genetic data, and perform appropriate cell biological and biochemical tests for validation. In this way, academic partnerships with clinical testing labs can further advance precision medicine for neurological disorders.

### ETHICAL COMPLIANCE

Human subjects. Participants were seen in the Center for Neurogenetics at Weill Cornell Medicine (WCM) and enrolled in neurogenetic studies under protocol 1402014809, approved by the WCM Investigational Review Board (IRB). Written informed consent was obtained from all subjects for genetic testing and/or skin biopsy.

## Data Availability

The data that support the findings of this study are available from the corresponding author upon reasonable request.
